# BMI Category and Survival in Incident Hemodialysis Patients: The Overweight Advantage in an Eastern European Cohort

**DOI:** 10.3390/jcm15082856

**Published:** 2026-04-09

**Authors:** Alexandru Catalin Motofelea, Nicu Olariu, Radu Pecingina, Luciana Marc, Lazar Chisavu, Flaviu Bob, Adelina Mihaescu, Adrian Apostol, Oana Schiller, Nadica Motofelea, Gheorghe Nicusor Pop, Andreea Crintea, Adalbert Schiller

**Affiliations:** 1 Department of Doctoral Studies, “Victor Babes” University of Medicine and Pharmacy Timisoara, 300041 Timisoara, Romania; alexandru.motofelea@umft.ro (A.C.M.); radu.pecingina@umft.ro (R.P.); nadica.motofelea@umft.ro (N.M.); 2 Centre for Molecular Research in Nephrology and Vascular Disease/MOL-NEPHRO-VASC, “Victor Babes” University of Medicine and Pharmacy Timisoara, 300041 Timisoara, Romania; marc.luciana@umft.ro (L.M.); chisavu.lazar@umft.ro (L.C.); bob.flaviu@umft.ro (F.B.); mihaescu.adelina@umft.ro (A.M.); schiller.adalbert@umft.ro (A.S.); 3 Department VII, Internal Medicine II, Division of Nephrology, “Victor Babes” University of Medicine and Pharmacy Timisoara, 300041 Timisoara, Romania; 4 County Emergency Hospital, L. Rebreanu Street, Nr. 156, 300723 Timisoara, Romania; 5 Department VII, Internal Medicine II, Discipline of Cardiology, “Victor Babes” University of Medicine and Pharmacy Timisoara, Eftimie Murgu Square No. 2, 300041 Timisoara, Romania; adrian.apostol@umft.ro; 6 Department of Cardiology, Pius Brinzeu Clinical Emergency County Hospital Timisoara, 300736 Timisoara, Romania; 7 B Braun Avitum Dialysis Centre, 300417 Timisoara, Romania; oana.leo67@gmail.com; 8 Department of Obstetrics and Gynecology, “Victor Babes” University of Medicine and Pharmacy Timisoara, Eftimie Murgu Square No. 2, 300041 Timisoara, Romania; 9 Center for Modeling Biological Systems and Data Analysis (CMSBAD), “Victor Babes” University of Medicine and Pharmacy Timisoara, 300041 Timisoara, Romania; 10 Department of Molecular Sciences, University of Medicine and Pharmacy “Iuliu Hațieganu”, 400349 Cluj-Napoca, Romania; crintea.andreea@umfcluj.ro

**Keywords:** hemodialysis, end-stage kidney disease, body mass index, mortality, overweight, obesity paradox

## Abstract

**Background**: Obesity, type 2 diabetes mellitus, and hypertension are increasingly prevalent components of metabolic syndrome and major contributors to cardiovascular disease and chronic kidney disease progression; however, in end-stage kidney disease an “obesity paradox” has been described, with higher body mass index (BMI) sometimes associated with improved survival on hemodialysis. **Material and methods**: This retrospective, single-center Eastern European cohort study aimed to characterize mortality and its causes around hemodialysis initiation in the contemporary era of cardiometabolic prevention and to test whether the obesity paradox persists at this high-risk transition. Adult patients initiating dialysis at the “Pius Brânzeu” Emergency Clinical Hospital (Timișoara, Romania) between January 2022 and December 2025 (*n* = 268; median age 66 years; 61% male; median eGFR 6.4 mL/min/1.73 m^2^) were analyzed using Kaplan–Meier methods and Cox regression, with comprehensive baseline clinical, laboratory, echocardiographic, medication, infection, and vascular access data; follow-up was obtained at 3, 6, 12, 24, and 36 months. **Results**: Late referral was common (61% < 3 months of nephrology follow-up), dialysis initiation was predominantly urgent (only 16% scheduled), and central venous catheters were the main access (81%), with substantial comorbidity burden (cardiovascular disease 71%, hypertension 90%) and frequent infections at initiation. BMI categories were non-obese (<25 kg/m^2^, 30%), overweight (25–29.9 kg/m^2^, 48%), and obese (≥30 kg/m^2^, 22%); diabetes prevalence rose with BMI (32% to 58%). Unadjusted mortality did not differ by BMI (19.8%, 18.8%, 15.3%; log-rank *p* = 0.622), yet multivariable Cox models showed overweight status independently reduced mortality (HR 0.22 at 3 months, 0.29 at 1 year, 0.31 at 3 years vs. non-obese), whereas obesity was not protective. Early mortality was driven mainly by age ≥ 65 years, while diabetes and chronic obstructive pulmonary disease predicted later mortality; longer pre-dialysis follow-up time was strongly protective (HR per year 0.70 at 3 years), and higher intact parathyroid hormone showed an inverse association with 1-year mortality. **Conclusions**: These findings show a modified obesity paradox at dialysis initiation in which moderate excess weight, but not obesity, is associated with improved adjusted survival, underscoring the clinical importance of earlier nephrology engagement and individualized nutritional and risk-factor management during the pre-dialysis and early dialysis periods.

## 1. Introduction

Obesity, type 2 diabetes mellitus (T2DM), and hypertension, key elements of metabolic syndrome, have shown alarming epidemiological trends over the last 50 years. By 2035, obesity prevalence is projected to double, reaching 24% compared to 12.5% in 2022 [1]. The global prevalence of T2DM is expected to rise to 9.5% by 2050, up from 5.9% in 2021 [2], and according to the World Health Organization [3], 33% of adults worldwide currently suffer from hypertension.

These components of metabolic syndrome exhibit complex interactions: up to 86% of all T2DM patients are either overweight or obese [4], up to 30% of obese individuals have T2DM [5], hypertension complicates the progression of T2DM in 50–75% of cases [5], and the prevalence of hypertension is 22.2% among the obese population and 15.5% among those who are overweight [6].

All elements of metabolic syndrome are major risk factors for the development and progression of cardiovascular disease (CVD) and mortality as well as of chronic kidney disease (CKD) and related comorbid conditions. In CKD (stages 1–5), in the CRIC cohort, the prevalence of metabolic syndrome was around 65%, being 87.5% among diabetics and 44.3% among nondiabetics and hypertension was the most frequent element of the metabolic syndrome [7]. In a recent meta-analysis, the high prevalence of metabolic syndrome persisted in end-stage kidney disease (ESKD), affecting up to 49% of cases [8]. Consequently, the modern phenotype of patients with ESKD initiating hemodialysis is characterized by failing kidney function, heavy CVD burden, and frequent comorbidities including T2DM, obesity, and hypertension, contributing to the very high mortality rate at hemodialysis initiation.

In recent years, complex prevention protocols have been implemented for both CVD risk and mortality reduction as well as prevention of CKD progression, including angiotensin II system interventions, SGLT2 inhibitors, more stringent blood pressure control, improved T2DM management, statins, and mineralocorticoid receptor antagonists. However, obesity remains a challenging issue. In the general population and in the early stages of CKD, obesity increases CVD risk, mortality, CKD risk, and disease progression. Conversely, in ESKD, the obesity paradox has been described, characterized by improved survival in hemodialysis-treated patients with obesity. Kalantar-Zadeh [9], demonstrated in a large retrospective hemodialysis cohort that higher BMI was associated with decreased risk of death, independent of age and hemodialysis vintage. This protective effect appeared to be more pronounced in younger incident hemodialysis patients (below 65 years of age), leading the authors to suggest increasing dry weight in these patients to reduce mortality [10]. Notably, the obesity paradox appears to be negated in incident patients who are both frail and obese, although frailty alone does not increase mortality in incident hemodialysis patients [11].

The aim of our Eastern European cohort study was to assess mortality and its causes at hemodialysis initiation in patients treated with these novel prevention protocols and to investigate whether the obesity paradox persists in this patient population.

## 2. Materials and Methods

### 2.1. Study Design and Setting

**Figure 1:**
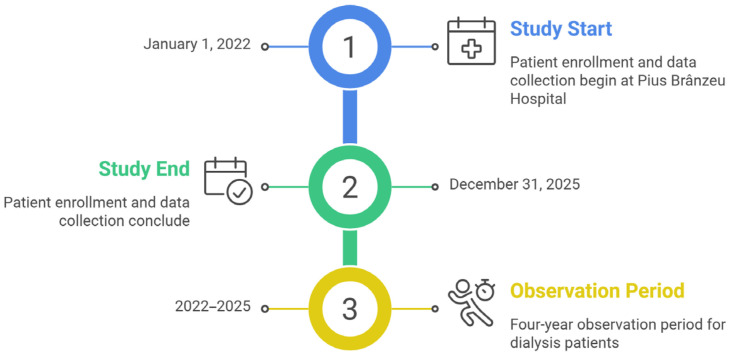
Study timeline and observation period. Numbers 1–3 indicate sequential study phases: (1) Study Start, (2) Study End, and (3) Observation Period; node colors correspond to each respective phase.

This retrospective cohort study was conducted at the “Pius Brânzeu” Emergency Clinical Hospital in Timișoara, Romania. The study employed a single-center, observational cohort design to evaluate patients who initiated dialysis treatment at our institution. The nephrology department of Pius Brânzeu Hospital serves as a regional referral center for patients with end-stage renal disease requiring renal replacement therapy. Patient enrollment and data collection spanned from January 2022 through December 2025, encompassing a four-year observation period (Figure 1).

### 2.2. Study Population

Patients were eligible for inclusion if they met all of the following criteria: initiated dialysis therapy at Pius Brânzeu Hospital between 1 January 2022, and 31 December 2025; age over 18 years, underwent complete evaluation in our nephrology department; and provided written informed consent to participate in the study. Patients were excluded from the study if they met any of the following: were initiated on dialysis at another healthcare facility and subsequently transferred to our center for follow-up only; did not provide written informed consent for study participation; or had incomplete medical records or insufficient data for analysis.

Of 335 patients who initiated dialysis at our institution during the study period, 67 were excluded: 7 did not provide informed consent, and 60 had incomplete medical records. The remaining 268 patients constituted the final study cohort. Given the retrospective nature of the study, a formal comparison of baseline characteristics between included and excluded patients was not feasible for those with incomplete records.

The study protocol was approved by the institutional ethics committee of Pius Brânzeu Hospital and was conducted in accordance with the Declaration of Helsinki and local regulatory requirements.

### 2.3. Data Collection and Outcomes

Data were extracted manually from electronic medical records and paper-based hospital files by a single trained investigator using a standardized data extraction form. The variables collected were: age, sex, body mass index, estimated glomerular filtration rate, serum creatinine, blood urea nitrogen, chronic kidney disease etiology, hypertension, diabetes mellitus, heart failure, chronic obstructive pulmonary disease, coronary artery disease, atrial fibrillation, hemoglobin, serum albumin, C-reactive protein, electrolytes, intact parathyroid hormone, serum calcium, serum phosphorus, left ventricular ejection fraction, valvular heart disease, infections at dialysis initiation, medications prior to dialysis, vascular access type, and hospitalization duration. Comorbidities were defined based on ICD-10 diagnostic codes recorded at hospital admission or established prior diagnoses documented in medical records. BMI was calculated as weight (kg) divided by height (m^2^), measured at dialysis initiation, and categorized according to WHO criteria: underweight (<18.5 kg/m^2^), normal weight (18.5–24.9 kg/m^2^), overweight (25.0–29.9 kg/m^2^), and obese (≥30.0 kg/m^2^). Given the absence of underweight patients in our cohort, the reference category was defined as normal weight (BMI < 25.0 kg/m^2^). Laboratory parameters were recorded as the first available value obtained at admission (baseline) and the last available value prior to discharge. The primary outcome was all-cause mortality. Follow-up data at 3, 6, 12, 24, and 36 months, including vital status and clinical parameters, were obtained from the dialysis centers to which patients were referred for maintenance hemodialysis. Vital status was ascertained through structured communication with the receiving dialysis centers and review of hospital mortality records. Patients lost to follow-up were censored at the date of last known contact. Missing values were not imputed; analyses were performed on available data.

### 2.4. Statistical Analysis

All statistical analyses were performed using Python 3.12 (Python Software Foundation). Data manipulation was conducted using pandas, statistical testing using SciPy, survival analyses using lifelines, and data visualization using matplotlib and seaborn. Continuous variables were assessed for normality using the Shapiro–Wilk test. Normally distributed data are presented as mean ± standard deviation and were compared using independent-samples t-tests for two-group comparisons or one-way analysis of variance (ANOVA) for multiple-group comparisons. Non-normally distributed data are presented as median with interquartile range [IQR] and were compared using the Mann–Whitney U test for two-group comparisons or the Kruskal–Wallis test for multiple-group comparisons. Categorical variables are presented as frequencies and percentages *n* (%) and were compared using the chi-square test. Fisher’s exact test was applied when expected cell counts were less than 5.

Survival analyses were conducted using the Kaplan–Meier method. Between-group comparisons were performed using the log-rank test. Survival probabilities were estimated at 1, 3, 6, 12, 24, and 36 months and are reported with 95% confidence intervals calculated using Greenwood’s formula. Survival curves were stratified by BMI categories. Cox proportional hazards regression was used to identify predictors of mortality. Univariable Cox models were fitted for all candidate variables. Variables with *p* < 0.10 in univariable analysis were entered into the multivariable model using backward selection. Effect estimates are reported as hazard ratios (HR) with 95% confidence intervals (CI). Model discrimination was assessed using the concordance index (C-index). The proportional hazards assumption was verified using Schoenfeld residuals. Multicollinearity among covariates was assessed using Variance Inflation Factors (VIF). All VIF values were below 2.0, indicating no meaningful collinearity among model predictors.

Endpoint analyses were performed at pre-specified timepoints (1, 3, 6, 12, 24, and 36 months) to compare cumulative mortality rates across BMI categories. Overall comparisons were performed using chi-square tests. Pairwise comparisons between BMI groups were conducted using chi-square or Fisher’s exact tests, as appropriate. A multivariable logistic regression model was constructed to identify independent predictors of mortality. Variables were selected based on clinical relevance and univariable screening (*p* < 0.10). The model was adjusted for demographics (age, sex), comorbidities (diabetes mellitus, heart failure, chronic obstructive pulmonary disease, atrial fibrillation), medications (statins, beta-blockers, ACE inhibitors, antidiabetic agents), vascular access type, laboratory parameters at admission, and anthropometric measures (BMI).

All statistical tests were two-tailed, and *p* < 0.05 was considered statistically significant. Analyses were conducted using complete-case methods, with the number of observations reported for each analysis. Missing data were not imputed. Results are reported in accordance with the STROBE guidelines for observational studies.

## 3. Results

A total of 268 patients initiated dialysis at Pius Brânzeu Hospital between January 2022 and December 2025. The cohort had a median age of 66 years (IQR: 54–72) with male predominance 164 (61%). At dialysis initiation, kidney function was severely compromised, with a median eGFR of 6.4 mL/min/1.73 m^2^ (IQR: 4.6–8.9).

Diabetic kidney disease emerged as the leading cause of chronic kidney disease 116 (43%), followed by hypertensive nephrosclerosis 53 (20%) and glomerulonephritis 51 (19%). The underlying etiology remained undetermined in 76 (28%) patients, predominantly due to late referral to nephrologist.

Late referral was a critical issue. The median time from first nephrology consultation to dialysis initiation was only 137 days (IQR: 18–1593 days). The majority of patients 158 (61%) had less than 3 months of nephrology follow-up, representing very late referrals. Another 58 (22.2%) were followed for 3 months to 2 years (late referral), while only 45 (17.2%) benefited from early referral with 2 or more years of follow-up.

Most patients, at admittance, presented urgent need of dialysis. The predominant indications were life-threatening complications: electrolyte imbalance resistant to conservative treatment 250 (93%), severe azotemia 223 (83%), and fluid overload 206 (77%). Uremic syndrome manifested as hemorrhagic complications was present in 66 (25%) and pericarditis 52 (19%). Metabolic acidosis was present in more than half 146 (54%). Only 43 (16%) patients had planned, (scheduled) dialysis initiation.

The burden of comorbidities was substantial, with cardiovascular disease affecting the majority of patients 189 (71%) and 179 (67%) experiencing some form of degenerative heart valve disorder. Specifically, mitral regurgitation was present in 185 (69%) of patients, tricuspid regurgitation in 154 (57%), and aortic regurgitation in 93 (35%). Heart failure was the predominant cardiovascular complication 147 (55%), accompanied by atrial fibrillation 53 (20%) and left ventricular hypertrophy 52 (19%). Nearly all patients had anemia 241 (90%), while hemorrhagic syndrome 42 (16%), malnutrition 35 (13%), and neuropathy 34 (13%) were also identified.

Hypertension was present in nearly all patients 242 (90%), with severe hypertension documented in the majority 77 (70%) of those with grading information. Coronary artery disease was evidenced in 78 patients (29%).

Beyond cardiovascular disease, other significant comorbidities included hyperuricemia 114 (43%), dyslipidemia 88 (33%), and chronic obstructive pulmonary disease 68 (25%). Hepatitis C viral infection was evidenced in 33 (12%) and Hepatitis B in 17 (6.3%) while 50 (19%) had a history of neoplasia.

Infections complicated nearly half of the patients admitted for initiation of HD. Urinary tract infections were most prevalent 129 (48%), followed by pneumonia 70 (26%) and catheter-related infections/sepsis 49 (18%). Additional infections included documented bacteremia 47 (18%), pyelonephritis 15 (5.6%), and COVID-19 17 (6.3%).

The type of vascular access reflected the urgency of initiation. The vast majority 216 (81%) started dialysis through a central venous catheter, indicating unplanned or emergency initiation. Only 112 (42%) had arteriovenous fistulas available.

Medication use before dialysis revealed extensive cardiovascular treatment needs. Antihypertensives were prescribed in 210 (78%) patients, predominantly calcium channel blockers 170 (63%) and beta-blockers 161 (60%). Loop diuretics were used in 160 (60%). Notably, renin-angiotensin system inhibitors were prescribed in only 36 (13.4%), likely reflecting concerns about hyperkalemia in advanced kidney disease. Other common medications included anticoagulants/antiplatelets 148 (55%), antidiabetic agents 125 (47%), and statins 94 (35%).

Hospital stays were prolonged, with a median of 14 days (IQR: 11–19). Nearly half of the cohort, 129 (48.1%), required hospitalizations exceeding 14 days, while 88 (33%) stayed 7–14 days, and only 48 (18%) had shorter stays under 7 days (Table 1).

BMI distribution revealed notable overweight/obesity prevalence in this dialysis cohort. Patients were stratified into three WHO categories: non-obese (<25 kg/m^2^) 81 (30%), overweight (25–29.9 kg/m^2^) 128 (48%), and obese (≥30 kg/m^2^) 59 (22%). Median BMI values were 23.3 kg/m^2^ (IQR: 22.0–24.2) for non-obese, 27.3 kg/m^2^ (IQR: 26.2–28.6) for overweight, and 31.5 kg/m^2^ (IQR: 30.8–34.0) for obese patients. Demographic differences across BMI categories were significant. Non-obese patients were younger (median 63 years, IQR: 48–71) compared to overweight patients (68 years, IQR: 57–73, *p* = 0.014). Male predominance was highest in the overweight group, 89 (70%), compared to the non-obese, 46 (57%), and obese groups, 29 (49%), though statistical significance was borderline (*p* = 0.065 and *p* = 0.365). Hematologic parameters showed BMI-dependent variations. Hemoglobin levels paralleled this pattern, with non-obese patients having lower values (9.1 g/dL) compared to overweight (9.8 g/dL, *p* = 0.048) and obese patients (9.7 g/dL, *p* = 0.163). Mean corpuscular volume was significantly higher in non-obese patients (87.8 fL) compared to obese patients (86.0 fL, *p* = 0.045). Metabolic alterations demonstrated clear BMI associations. Uric acid levels increased progressively with BMI: non-obese 6.8 mg/dL, overweight 7.2 mg/dL, obese 8.1 mg/dL (*p* = 0.001 for non-obese vs. obese). Intact parathyroid hormone levels were significantly elevated in obese patients (385.2 pg/mL) compared to overweight patients (239.2 pg/mL, *p* = 0.034), with non-obese patients showing intermediate values (304.3 pg/mL). Calcium levels were significantly higher in overweight patients compared to non-obese (*p* = 0.038), while phosphorus was significantly elevated in obese versus non-obese patients (*p* = 0.043). Lipid profiles revealed an inverse relationship with BMI. Total cholesterol was significantly higher in non-obese patients (107.0 mg/dL) compared to overweight (76.0 mg/dL, *p* = 0.011) and obese patients (70.5 mg/dL, *p* = 0.019). LDL cholesterol showed a similar trend (non-obese: 103.0 mg/dL vs. overweight: 84.0 mg/dL, *p* = 0.051). HDL cholesterol demonstrated borderline significance, with non-obese patients having higher levels (38.5 mg/dL) than overweight (34.4 mg/dL, *p* = 0.051) and obese patients (34.6 mg/dL, *p* = 0.053). These findings suggest possible malnutrition-inflammation-cachexia syndrome in lower BMI patients. Inflammatory and nutritional markers were comparable across BMI groups. CRP, albumin, transferrin, and fibrinogen showed no significant differences, indicating similar inflammatory and nutritional states regardless of BMI. Neutrophil percentage showed a trend toward higher values in non-obese patients (73.3%) compared to overweight patients (68.8%, *p* = 0.060). Diabetes mellitus prevalence increased significantly with BMI. The proportion nearly doubled from non-obese 26 (32%) to overweight 56 (44%) and obese 34 (58%, *p* = 0.003 for non-obese vs. obese). Antidiabetic medication use paralleled this gradient: non-obese 28 (35%), overweight 62 (48%, *p* = 0.040), obese 35 (59%, *p* = 0.003). HbA1c levels showed a non-significant trend toward higher values with increasing BMI. Cardiovascular parameters and other comorbidities showed no BMI-related differences. Left ventricular ejection fraction, valvular disease prevalence, hypertension, heart failure, coronary artery disease, and anemia were comparable across BMI categories.

Mortality rates demonstrated a non-significant trend favoring higher BMI. Total mortality was 16 (20%) in non-obese, 24 (19%) in overweight, and 9 (15%) in obese patients (*p* = 0.780 and *p* = 0.498) (Table 2).

Overweight patients demonstrated the lowest mortality at 3 months (37.5% vs. 56.2% non-obese vs. 77.8% obese, *p* = 0.105) and 12 months (58.3% vs. 75.0% vs. 77.8%, *p* = 0.415). Near-complete mortality convergence occurred by 36 months (93.8–100%).

**Figure 2:**
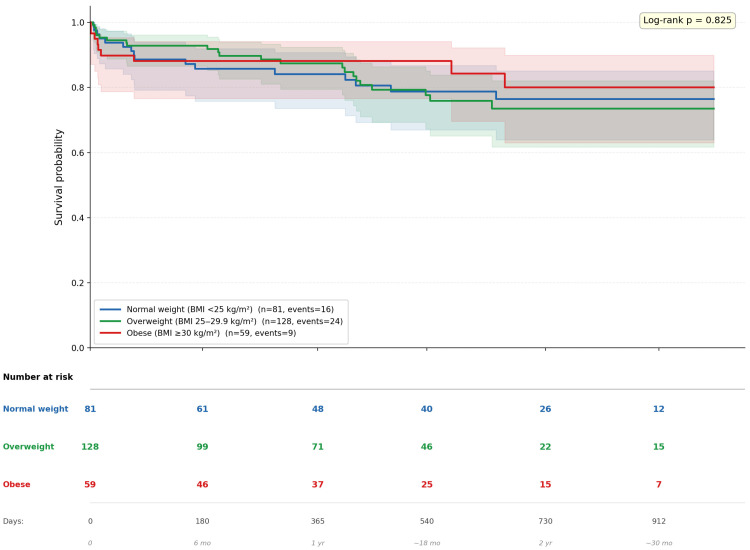
Kaplan–Meier survival curves stratified by BMI category at dialysis initiation.

Overall mortality was numerically lowest in obese patients (15.3%) compared to overweight (18.8%) and non-obese patients (19.8%), though differences were not statistically significant (*p* = 0.825). The lower early mortality in overweight patients and lower overall mortality in obese patients suggest a possible obesity paradox in this dialysis cohort (Figure 2).

In this cohort of 268 incident dialysis patients, overweight status (BMI 25–29.9 kg/m^2^) was independently associated with reduced mortality risk across all time horizons, with hazard ratios of 0.22 (95% CI, 0.05–0.95) at 3 months, 0.29 (95% CI, 0.09–0.93) at 1 year, and 0.31 (95% CI, 0.12–0.81) at 3 years compared with normal-weight patients. This protective association was not observed among patients with obesity (BMI ≥ 30 kg/m^2^), whose mortality risk did not differ significantly from that of normal-weight patients at any time point. The temporal pattern of risk factors revealed distinct phases of vulnerability. Advanced age (≥65 years) conferred the highest relative mortality risk in the early post-dialysis initiation period (HR, 11.77; 95% CI, 1.38–100.18 at 3 months), though this association attenuated substantially over time (HR, 3.14; 95% CI, 1.31–7.53 at 3 years). Conversely, diabetes mellitus and chronic obstructive pulmonary disease demonstrated delayed effects, becoming statistically significant predictors only at 3 years (HR, 2.75 [95% CI, 1.05–7.20] and HR, 2.49 [95% CI, 1.05–5.90], respectively). This pattern suggests that immediate post-dialysis mortality is driven primarily by acute physiological vulnerability and frailty, whereas longer-term mortality reflects the cumulative burden of comorbid conditions.

The protective association between longer time to dialysis initiation and mortality (HR per year, 0.70; 95% CI, 0.58–0.85 at 3 years) underscores the potential benefit of strategies to preserve residual kidney function and delay dialysis initiation when clinically appropriate. Each additional year of preserved kidney function was associated with a 30% reduction in 3-year mortality risk.

The inverse association between intact parathyroid hormone levels and mortality (HR per 100 pg/mL, 0.62; 95% CI, 0.39–0.98 at 1 year) warrants cautious interpretation. While this may reflect reverse epidemiology—wherein higher PTH levels mark better nutritional status or less aggressive suppression, further investigation is needed to determine whether this represents a causal pathway or residual confounding (Table 3).

## 4. Discussion

Our study challenges the broad generalization that higher BMI protects hemodialysis patients by showing that this effect is specifically attributable to overweight status (BMI 25–29.9 kg/m^2^) rather than obesity (BMI ≥ 30 kg/m^2^). Cox proportional hazards regression revealed that the protective effect of higher BMI was specifically attributable to overweight status (BMI 25–29.9 kg/m^2^) rather than obesity (BMI ≥ 30 kg/m^2^). Overweight patients demonstrated significant mortality protection across all time horizons: HR 0.22 (95% CI: 0.05–0.95, *p* = 0.042) at 3 months, HR 0.29 (95% CI: 0.09–0.93, *p* = 0.037) at 1 year, and HR 0.31 (95% CI: 0.12–0.81, *p* = 0.017) at 3 years. In contrast, obesity conferred no significant protection at any timepoint (3-month HR 1.13, *p* = 0.880; 1-year HR 1.33, *p* = 0.716; 3-year HR 1.51, *p* = 0.541).

Overall mortality rates showed no significant differences in unadjusted analysis (non-obese: 19.8%, overweight: 18.8%, obese: 15.3%, *p* = 0.780), nor did Kaplan–Meier survival curves (log-rank *p* = 0.825). This discrepancy between adjusted and unadjusted analyses underscores that the protective effect of overweight status emerges only after controlling for confounding variables.

Our findings partially align with the extensive literature documenting the obesity paradox in hemodialysis populations. Earlier large observational studies broadly demonstrated that higher BMI at dialysis initiation is associated with improved survival [12,13,14,15], and this inverse relationship has been replicated across different ethnicities [16].

However, most of these studies treated higher BMI as a single category without systematically distinguishing between overweight and obesity. More recent data suggest that this survival advantage is not uniformly distributed across all BMI categories. In the European COSMOS cohort of 6296 hemodialysis patients, the protective association of weight change with mortality was notably less apparent among obese compared to non-obese patients [17], and a nationwide Korean registry analysis of over 10,000 hemodialysis patients demonstrated that the beneficial effect of obesity disappeared entirely after 7 years of follow-up, with young obese patients even experiencing increased long-term mortality [18]. Similarly, Ertilav et al. [19] reported that in 3252 prevalent hemodialysis patients followed for 7 years, BMI was not a mortality predictor in patients with dialysis duration exceeding 76 months, and that the protective effect of higher BMI was most pronounced in inflamed patients, with a 5-fold short-term mortality increase in inflamed patients in the lowest versus the highest BMI quartile.

The protective effect of overweight status in our Cox regression model (HR 0.22–0.31) is consistent with the general direction of the obesity paradox literature [20,21]. However, unlike the linear models reported in these meta-analyses, suggesting a continuous 3–4% mortality reduction per 1 kg/m^2^ increase, our results show a categorical pattern: overweight was significantly protective, whereas obesity conferred no survival advantage (HR 1.13–1.51, all *p* > 0.5). This is supported by nonlinear patterns acknowledged within both meta-analyses, where Rahimlu et al. [21] observed increasing mortality above BMI 30 kg/m^2^ and Ladhani et al. noted that underweight status drove much of the apparent benefit of higher BMI [20,21]. These findings reinforce the importance of distinguishing between overweight and obesity in dialysis outcome studies.

Our finding that obesity (BMI ≥ 30 kg/m^2^) conferred no significant protection (HR 1.13–1.51, all *p* > 0.5) aligns with studies reporting U- or J-shaped relationships, with increased mortality at both extremes of BMI [22,23]. This distinction suggests that the obesity paradox in dialysis populations may be more accurately characterized as an “overweight paradox,” with moderate excess weight rather than obesity per se driving the survival advantage reported in previous studies. The most recent meta-analysis by Kusuma et al. [24], pooling 15 studies involving 381,580 hemodialysis patients, further underscores that the obesity paradox is measurement-dependent: while BMI-defined obesity was associated with lower mortality (RR 0.73, 95% CI: 0.70–0.76), abdominal obesity measured by waist circumference or waist-to-hip ratio increased mortality risk (RR 1.35, 95% CI: 1.01–1.80), and bioelectrical impedance analysis similarly demonstrated increased mortality in obese patients (RR 1.22, 95% CI: 1.05–1.41). This interpretation is further supported by our multicenter analysis of 679 maintenance hemodialysis patients, in which obesity was independently associated with increased all-cause mortality (HR 1.411, *p* = 0.045), whereas overweight status did not confer a clear survival disadvantage over normal weight [25].

This suggests that moderate excess weight, rather than obesity per se, drives the survival benefit observed in dialysis populations.

Our study adds to this literature by demonstrating a modified obesity paradox specifically at dialysis initiation, a critical transition period associated with high mortality risk. The 13.1% 1-year mortality rate observed in our cohort underscores the vulnerability of incident dialysis patients, making identification of protective factors particularly important for this population. Similar protective associations have been reported in peritoneal dialysis patients as well [26].

Although our study examined baseline BMI rather than weight trajectories, existing literature emphasizes the prognostic importance of weight changes during early dialysis. Kalantar-Zadeh et al. [27] demonstrated that progressively worsening weight loss was significantly associated with poorer survival and higher cardiovascular mortality in a large cohort of maintenance hemodialysis patients. Chazot et al. [28] showed that patients experiencing weight loss exceeding 5.8% in the first year had significantly lower survival compared to those maintaining stable weight.

Chang et al. [29] provided particularly relevant insights by characterizing weight change trajectories in incident dialysis patients. They reported that postdialysis weights rapidly decreased and reached a nadir at the 5th month of dialysis, with average declines of 2% from baseline. Importantly, patients experiencing 6% or greater weight loss during the first 5 months had a 14% increased mortality hazard, while those achieving 2–6% weight gain during months 5–12 demonstrated 9% lower mortality hazards [29]. These findings underscore that the obesity paradox extends beyond baseline BMI to encompass dynamic weight changes during the critical early dialysis period.

Several recent studies have consistently described that short-term weight gains and losses were associated with lower and higher mortality risk, respectively, in the first 6 months of dialysis [9,17,30].

Our finding that overweight patients demonstrated particularly low early mortality (37.5% of deaths occurring by 3 months vs. 56.2% in non-obese and 77.8% in obese) may reflect the importance of metabolic reserve during the initial dialysis adaptation period. The significant protective effect of longer pre-dialysis follow-up time (HR 0.70 per year, *p* < 0.001 at 3 years) further supports the concept that patients entering dialysis with better preserved nutritional and metabolic status, whether reflected by moderate BMI, longer nephrology care, or both, demonstrate improved survival outcomes.

Patients with moderate excess body mass may better tolerate hemodialysis-associated hemodynamic stress. Hypertension was nearly universal in our cohort (90%), and previous studies have reported lower mortality with higher blood pressure in heart failure patients [31,32].

Lower BMI may reflect underlying frailty and cachexia rather than an independent mortality pathway. Fitzpatrick et al. showed that frailty increases mortality risk only in the presence of obesity, not in non-obese hemodialysis patients [11], and Szeto et al. confirmed a similar interaction between frailty and central obesity in peritoneal dialysis [33] suggesting that it is the frail-obese phenotype, rather than frailty alone, that abolishes the survival advantage attributed to higher BMI.

The interaction between BMI and diabetes merits specific discussion, as diabetes prevalence in our cohort increased with BMI (32% non-obese, 44% overweight, 58% obese, *p* = 0.003). El-Khoury et al. [34] reported that obesity was associated with lower mortality in heart failure patients with diabetes. Costanzo et al. [35] demonstrated lower mortality in overweight patients with type 2 diabetes, though obesity showed no significant benefit—a pattern similar to our findings. Afghahi et al. [36] found that obese individuals were less likely to die except when receiving dialysis, contrasting somewhat with our results. Park et al. [37] showed that the U-shaped BMI-mortality relationship was less significant in diabetic patients with acute coronary syndrome.

In our cohort, diabetes demonstrated a delayed mortality effect, reaching significance only at 3 years (HR 2.75, *p* = 0.039) but not earlier (1-year HR 2.39, *p* = 0.136). This contrasts with the dominant early effect of advanced age (HR 11.77 at 3 months), suggesting diabetic patients face cumulative rather than immediate risk.

### 4.1. Clinical Implications

The temporal pattern of risk factors enables targeted interventions. Advanced age dominated early mortality (HR 11.77 at 3 months, attenuating to HR 3.14 at 3 years), warranting intensive early monitoring in elderly patients. Diabetes and COPD demonstrated delayed effects (both significant only at 3 years: HR 2.75 and HR 2.49, respectively), emphasizing sustained comorbidity management.

Overweight status (HR 0.22–0.31) and longer pre-dialysis follow-up (HR 0.70 per year) were protective. Weight reduction strategies may be contraindicated in overweight patients (BMI 25–29.9 kg/m^2^). However, obesity conferred no protection, suggesting body composition optimization rather than further weight gain for obese patients.

Current guidelines recommend protein intake of 1.0–1.2 g/kg/day for hemodialysis patients. Our findings support nutritional optimization beginning in the pre-dialysis period. The 61% prevalence of very late referral (<3 months) in our cohort may limit time for adequate nutritional preparation. Comparable inflammatory markers across BMI categories suggest the protective effect of overweight status operates through mechanisms beyond simple nutritional adequacy.

### 4.2. Strengths

This study offers several methodological strengths. The single-center design ensured consistent protocols for dialysis initiation, baseline evaluation, and clinical management. All patients were initiated on dialysis at our institution, eliminating referral bias from transfers. Comprehensive data collection captured detailed comorbidity profiles, laboratory panels, cardiovascular assessments, and medication histories, allowing robust multivariable adjustment.

The Cox proportional hazards analysis at multiple timepoints (3, 12, and 36 months) enabled assessment of temporal dynamics in risk factors, revealing the important distinction between early predictors (age) and late predictors (diabetes, COPD).

### 4.3. Limitations

The single-center, retrospective design limits generalizability to other populations, healthcare settings, and geographic regions. The relatively small sample size (268 patients, 49 deaths) resulted in wide confidence intervals for some estimates, particularly at 3 months (age ≥ 65 years: HR 11.77, 95% CI: 1.38–100.18). The lack of statistically significant differences in Kaplan–Meier analysis (log-rank *p* = 0.622) despite significant findings in adjusted Cox regression highlights limited power for unadjusted comparisons.

BMI cannot distinguish muscle from fat mass or assess body fat distribution. The differential effects of overweight versus obesity observed underscore the need for body composition assessment in future studies. The study period (2022–2025) included COVID-19 pandemic effects (6.3% prevalence at initiation), potentially limiting generalizability.

The observational design cannot establish causality. Unmeasured confounding by frailty, physical function, socioeconomic status, or medication adherence may influence observed associations. Variable follow-up duration and near-complete mortality convergence by 36 months (93.8–100% across BMI categories) may reflect survival bias at later timepoints.

It is also important to note that our study period overlaps with the publication of several practice-changing trials including CREDENCE (2019) [38], DAPA-CKD (2020) [39], EMPA-KIDNEY (2022) [40], and STOP-ACEi (2022) [41] that established SGLT2 inhibitors as a cornerstone of cardiorenal protection and confirmed the safety of continuing renin–angiotensin system inhibitors in advanced CKD. Given this temporal overlap, these guideline-changing interventions would have only partially influenced the management of our cohort, which may partly explain the apparently low utilization of ACE inhibitors observed in our population. With 49 observed deaths, the events-per-variable ratio in our multivariable Cox models approached but did not consistently meet the recommended threshold of ≥10, raising the possibility of model overfitting and contributing to the wide confidence intervals observed for some estimates, particularly at 3 months. These findings should be interpreted with appropriate caution and require validation in larger prospective cohorts. Future studies in incident hemodialysis patients should prospectively incorporate objective body composition assessment, including BIA-derived phase angle, lean tissue index, and fat tissue index, to disentangle the contributions of muscle mass, adiposity, and fluid status to the BMI-mortality relationship. Such approaches would allow testing of whether the protective effect of overweight status is mediated primarily through muscle mass preservation, through metabolic reserve capacity, or through both pathways simultaneously.

## 5. Conclusions

In this cohort of 268 incident hemodialysis patients, overweight but not obesity was independently protective across all time horizons, supporting an “overweight paradox” rather than a broad obesity paradox. Risk factors followed distinct temporal patterns, with age dominating early and diabetes and COPD emerging as late predictors. Longer pre-dialysis nephrology follow-up was independently protective. These findings support differentiating overweight from obesity in clinical decision-making and incorporating body composition assessment beyond BMI.

## Figures and Tables

**Table 1:** Baseline Characteristics of Patients at Dialysis Initiation.

Demographics		Comorbidities	
**Age, years (Median, IQR)**	66 (54, 72)	Hypertension	242 (90%)
**Sex**		Grade I (mild)	6 (5.5%)
Male	164 (61%)	Grade II (moderate)	27 (25%)
Female	104 (39%)	Grade III (severe)	77 (70%)
**kidney function at initiation**			
eGFR, mL/min/1.73 m^2^ (Median, IQR)	6.4 (4.6, 8.9)	Heart failure	164 (61%)
		Coronary artery disease	78 (29%)
**Primary etiology of CKD**			
Hypertensive nephrosclerosis	53 (20%)	Acute coronary syndrome	32 (12%)
Diabetic kidney disease	116 (43%)	Mitral regurgitation	185 (69%)
Glomerulonephritis	51 (19%)	Aortic regurgitation	93 (35%)
Polycystic kidney disease	19 (7.1%)	Tricuspid regurgitation	154 (57%)
Chronic tubulointerstitial nephropathy	16 (6.0%)	Aortic stenosis	15 (5.6%)
Single surgical kidney	9 (3.4%)	Cardiovascular disease	189 (71%)
Other *	7 (2.6%)	Valvulopathy	179 (67%)
Undetermined	76 (28%)	Heart failure	147 (55%)
**Reasons for dialysis initiation**			
**Electrolyte imbalance**	250 (93%)	Atrial fibrillation	53 (20%)
**Increased azotemia**	223 (83%)	Left ventricular hypertrophy	52 (19%)
**Fluid retention**	206 (77%)	Pericarditis	52 (19%)
**Metabolic acidosis**	146 (54%)	Coronary artery disease	18 (6.7%)
**Hemorrhagic syndrome**	66 (25%)	Cardiomyopathy	16 (6.0%)
**Pericarditis**	52 (19%)	Anemia	240 (90%)
**Scheduled initiation**	43 (16%)	Dyslipidemia	88 (33%)
**Unmanageable hypertension**	11 (4.1%)	Hyperuricemia	114 (43%)
**Time from first nephrology visit**			
**Days (Median, IQR)**	137 (18, 1593)	COPD/Respiratory disease	68 (25%)
**Months (Median, IQR)**	5 (1, 53)	Cerebrovascular disease (stroke/TIA)	26 (9.7%)
**Years (Median, IQR)**	0.4 (0.0, 4.4)	Gastritis/Peptic ulcer	12 (4.5%)
**Follow-up before initiation**		Hypothyroidism	9 (3.4%)
**<3 months (very late referral)**	158 (61%)	Hepatitis B	17 (6.3%)
**3 months–2 years (late referral)**	58 (22.2%)	Hepatitis C	33 (12%)
≥**2 years (early referral)**	45 (17.2%)	Neoplasia	50 (19%)
**Other complications at initiation**		**Infections at dialysis initiation**	
**Anemia**	241 (90%)	Urinary tract infection	129 (48%)
**Hemorrhage**	42 (16%)	Pneumonia	70 (26%)
**Malnutrition**	35 (13%)	Catheter infection/Sepsis	49 (18%)
**Neuropathy**	34 (13%)	Bacterial infection (documented)	47 (18%)
**Cerebrovascular event**	26 (9.7%)	Pyelonephritis	15 (5.6%)
**Thrombosis/Pulmonary embolism**	11 (4.1%)	COVID-19	17 (6.3%)
**Hyperparathyroidism**	3 (1.1%)	Endocarditis	12 (4.5%)
**Hypoglycemia**	2 (0.7%)	Skin/Soft tissue infection	8 (3.0%)
**Vascular access**			
**Arteriovenous fistula (AVF)**	112 (42%)	Central venous catheter (CVC)	216 (81%)
**Prior treatment before dialysis**			
**ACE inhibitors/ARBs**	36 (13.4%)	Antihypertensives	210 (78%)
**Calcium channel blockers**	170 (63%)	Anticoagulants/Antiplatelets	148 (55%)
**Beta-blockers**	161 (60%)	**Antiplatelets**	75 (28%)
**Loop diuretics**	160 (60%)	Anticoagulants	65 (24%)
**Potassium-sparing diuretics**	9 (3.4%)	Antiarrhythmics	10 (3.7%)
**Statins**	94 (35%)	SGLT2 inhibitors	2 (0.7%)
**Iron supplementation**	76 (28%)	Antidiabetic treatment	125 (47%)
**Corticosteroids**	49 (18%)	Antianginal agents	28 (10%)
**Erythropoietin**	45 (17%)	Immunosuppressants	13 (4.9%)
**Hospitalization**			
**Length of stay, days (Median, IQR)**	14 (11, 19)	<7 days	48 (18%)
		7–14 days	88 (33%)
		>14 days	129 (48.1%)

* Other etiologies include: Multiple myeloma, Lupus nephritis, Obstructive uropathy, Vascular nephropathy. Primary etiology percentages sum to >100% as some patients had multiple contributing etiologies. Values are presented as median (Q1, Q3) for continuous variables and *n* (%) for categorical variables. Abbreviations: CKD = Chronic Kidney Disease; eGFR = estimated Glomerular Filtration Rate; TIA = Transient Ischemic Attack; COPD = Chronic Obstructive Pulmonary Disease; ACE = Angiotensin-Converting Enzyme; ARB = Angiotensin Receptor Blocker; SGLT2 = Sodium-Glucose Cotransporter-2; CVC = Central Venous Catheter; AVF = Arteriovenous Fistula. Bold entries indicate major category headers.

**Table 2:** Comparison of Patient Characteristics by BMI Category.

Clinical Characteristics	Total (*n* = 268)	Non-Obese (<25 kg/m^2^) *n*= 81 (30%)	Overweight (25–29.9 kg/m^2^) *n*= 128 (48%)	Obese (≥30 kg/m^2^) *n* = 59 (22%)	*p*-Value (Non-Obese vs. Overweight)	*p*-Value (Non-Obese vs. Obese)
Age, years	66 (54–72)	63 (48–71)	68 (57–73)	64 (55–71)	0.014	0.366
Male sex, *n* (%)	164 (61%)	46 (57%)	89 (70%)	29 (49%)	0.065	0.365
BMI, kg/m^2^	27.0 (23.8–29.9)	23.3 (22.0–24.2)	27.3 (26.2–28.6)	31.5 (30.8–34.0)	—	—
Hemoglobin, g/dL	9.6 (8.4–10.8)	9.1 (8.2–10.6)	9.8 (8.6–10.8)	9.7 (8.5–11.0)	0.048	0.163
Ferritin, ng/mL	269.5 (139.7–495.5)	301.6 (138.1–551.1)	259.9 (143.5–475.0)	241.7 (127.8–420.1)	0.603	0.306
Creatinine, mg/dL	8.0 (5.8–9.9)	8.4 (5.5–9.7)	7.7 (5.9–9.9)	8.2 (6.5–10.1)	0.641	0.368
Uric acid, mg/dL	7.2 (6.2–8.6)	6.8 (6.2–8.3)	7.2 (6.1–8.4)	8.1 (7.2–9.9)	0.527	0.001
eGFR, mL/min/1.73 m^2^	6.5 (4.6–9.1)	6.3 (4.5–9.6)	7.0 (4.9–9.1)	5.8 (4.5–7.7)	0.573	0.078
Calcium, mg/dL	8.2 (7.4–8.8)	8.0 (7.0–8.7)	8.3 (7.6–8.8)	8.0 (7.0–8.7)	0.038	0.713
Phosphorus, mg/dL	6.2 (5.2–7.6)	6.1 (5.1–7.9)	6.0 (5.0–7.3)	6.6 (5.9–7.8)	0.368	0.043
Ca × Phos product, mg^2^/dL^2^	49.7 (40.9–60.7)	49.2 (39.4–60.5)	49.2 (41.8–60.0)	53.4 (42.3–61.5)	0.685	0.254
iPTH, pg/mL	285.0 (153.1–492.8)	304.3 (181.9–590.5)	239.2 (136.4–392.9)	385.2 (264.9–608.9)	0.034	0.094
Glucose, mg/dL	105.0 (91.0–135.0)	103.0 (89.0–138.0)	104.0 (92.2–127.8)	112.0 (94.8–148.0)	0.731	0.143
HbA1c, %	5.2 (4.8–6.2)	5.0 (4.7–6.2)	5.3 (4.9–6.1)	5.5 (4.8–6.2)	0.282	0.319
Total cholesterol, mg/dL	83.0 (44.0–134.5)	107.0 (52.0–163.0)	76.0 (40.0–116.5)	70.5 (39.3–117.2)	0.011	0.019
LDL cholesterol, mg/dL	88.0 (65.0–113.5)	103.0 (67.2–118.8)	84.0 (61.0–104.0)	82.0 (67.0–117.0)	0.051	0.414
HDL cholesterol, mg/dL	35.9 (29.3–42.1)	38.5 (30.5–44.3)	34.4 (28.4–40.8)	34.6 (29.2–39.7)	0.051	0.053
Triglycerides, mg/dL	133.0 (97.0–170.5)	131.0 (97.0–172.0)	134.0 (95.2–163.8)	133.0 (107.8–183.5)	0.937	0.302
Albumin, g/dL	3.7 (3.2–4.1)	3.7 (3.1–4.2)	3.7 (3.2–4.0)	3.6 (3.3–4.1)	0.943	0.723
Transferrin, mg/dL	170.5 (129.5–199.0)	172.0 (132.0–192.0)	167.0 (123.5–202.5)	172.5 (135.5–195.0)	0.995	0.740
CRP, mg/L	19.5 (6.1–47.4)	19.8 (5.0–47.6)	18.8 (6.5–44.9)	21.6 (8.9–60.0)	0.589	0.275
Neutrophils, %	70.8 (62.6–77.6)	73.3 (64.0–80.1)	68.8 (61.8–76.7)	71.2 (62.2–76.2)	0.060	0.296
LVEF, %	51.0 (43.0–55.0)	50.0 (45.0–55.0)	52.0 (42.5–55.0)	50.0 (42.5–53.0)	0.704	0.345
**Comorbidities and outcomes**						
Hypertension, *n* (%)	242 (90%)	74 (91%)	112 (88%)	56 (95%)	0.396	0.328
Diabetes mellitus, *n* (%)	116 (43%)	26 (32%)	56 (44%)	34 (58%)	0.099	0.003
Heart failure, *n* (%)	164 (61%)	47 (58%)	77 (60%)	40 (68%)	0.788	0.208
Coronary artery disease, *n* (%)	78 (29%)	18 (22%)	39 (30%)	21 (36%)	0.205	0.070
Anemia, *n* (%)	240 (90%)	77 (95%)	112 (88%)	51 (86%)	0.082	0.056
Statins, *n* (%)	94 (35%)	27 (33%)	42 (33%)	25 (42%)	0.945	0.243
Total Mortality, *n* (%)	49 (18%)	16 (20%)	24 (19%)	9 (15%)	0.869	0.498

BMI Categories (WHO Classification): Non-obese (<25 kg/m^2^); Overweight (25–29.9 kg/m^2^); Obese (≥30 kg/m^2^). Values are presented as median (Q1–Q3) for continuous variables and *n* (%) for categorical variables. *p*-values calculated using Mann–Whitney U test for continuous variables and Chi-square test or Fisher’s exact test for categorical variables. LDL, HDL, and neutrophils showed borderline significance (*p* = 0.051, 0.051, 0.053, and 0.060, respectively). Abbreviations: BMI = Body Mass Index; eGFR = estimated Glomerular Filtration Rate; Ca × Phos product = Calcium-Phosphorus Product; iPTH = intact Parathyroid Hormone; HbA1c = Hemoglobin A1c; LDL = Low-Density Lipoprotein; HDL = High-Density Lipoprotein; CRP = C-Reactive Protein; LVEF = Left Ventricular Ejection Fraction.

**Table 3:** Cox Proportional Hazards Regression Analysis for All-Cause Mortality in Dialysis Patients.

Variable	3-Month Mortality		1-Year Mortality		3-Year Mortality	
Outcome measure	HR (95% CI)	*p*-value	HR (95% CI)	*p*-value	HR (95% CI)	*p*-value
N/Events	27/268		35/268		49/268	
Age ≥ 65 years	11.77 (1.38–100.18)	0.024 *	6.10 (1.57–23.75)	0.009 **	3.14 (1.31–7.53)	0.010 *
Overweight (BMI 25–29.9) vs. Normal	0.22 (0.05–0.95)	0.042 *	0.29 (0.09–0.93)	0.037 *	0.31 (0.12–0.81)	0.017 *
Obese (BMI ≥ 30) vs. Normal	1.13 (0.23–5.54)	0.88	1.33 (0.28–6.33)	0.716	1.51 (0.40–5.73)	0.541
COPD/Respiratory disease	2.16 (0.59–7.89)	0.245	1.53 (0.55–4.30)	0.418	2.49 (1.05–5.90)	0.037 *
Albumin at admission (per g/dL)	0.44 (0.12–1.68)	0.232	0.33 (0.11–1.06)	0.062	0.46 (0.19–1.13)	0.092
Diabetes mellitus	1.30 (0.35–4.77)	0.696	2.39 (0.76–7.48)	0.136	2.75 (1.05–7.20)	0.039 *
Heart failure	0.80 (0.15–4.16)	0.791	1.02 (0.22–4.74)	0.982	1.50 (0.48–4.67)	0.488
Time to dialysis initiation (per year)	0.84 (0.66–1.06)	0.138	0.73 (0.58–0.92)	0.008 **	0.70 (0.58–0.85)	<0.001 ***
Ca × P product > 55 mg^2^/dL^2^	1.29 (0.16–10.25)	0.807	1.01 (0.17–5.85)	0.995	1.10 (0.31–3.87)	0.882
Phosphorus (per mg/dL)	0.82 (0.47–1.42)	0.481	0.89 (0.56–1.40)	0.608	0.97 (0.68–1.39)	0.878
iPTH (per 100 pg/mL)	0.74 (0.48–1.15)	0.179	0.62 (0.39–0.98)	0.040 *	0.71 (0.54–0.94)	0.017 *

HR, hazard ratio; CI, confidence interval; BMI, body mass index; COPD, chronic obstructive pulmonary disease; Ca × P, calcium-phosphorus; iPTH, intact parathyroid hormone. Reference category for BMI: normal weight (BMI 18.5–24.9 kg/m^2^). Time to dialysis initiation represents pre-dialysis nephrology follow-up duration.* *p* < 0.05; ** *p* < 0.01; *** *p* < 0.001.

## Data Availability

The data presented in this study are available upon reasonable request from the corresponding author. The data are not publicly available due to privacy and ethical restrictions.
